# Co-Fermentation of Agri-Food Residues Using a Co-Culture of Yeasts as a New Bioprocess to Produce 2-Phenylethanol

**DOI:** 10.3390/molecules28145536

**Published:** 2023-07-20

**Authors:** Mariana Valdez Castillo, Satinder Kaur Brar, Sonia Arriaga, Jean-François Blais, Michèle Heitz, Antonio Avalos Ramirez

**Affiliations:** 1 Institut National de la Recherche Scientifique, Centre-Eau Terre Environnement, 490, Rue de la Couronne, Québec City, QC G1K 9A9, Canada; mariana.valdez_castillo@inrs.ca (M.V.C.); jean-francois.blais@inrs.ca (J.-F.B.); 2 Département de Génie Chimique et de Génie Biotechnologique, Faculté de Génie, Université de Sherbrooke, 2500 Boulevard de l’Université, Sherbrooke, QC J1K 2R1, Canada; michele.heitz@usherbrooke.ca; 3 Centre National en Électrochimie et en Technologies Environnementales, 2263, Avenue du Collège, Shawinigan, QC G9N 6V8, Canada; 4 Department of Civil Engineering, Lassonde School of Engineering, York University, Toronto, ON M3J 1P3, Canada; 5 Instituto Potosino de Investigación Científica y Tecnológica (IPICyT), División de Ciencias Ambientales, Camino a la Presa San José 2055, Lomas 4a Sección, San Luis Potosi CP 78216, Mexico; sonia@ipicyt.edu.mx

**Keywords:** whey treatment, residue valorization, yeast fermentation, 2-phenylethanol, stirred tank bioreactor

## Abstract

Whey is a dairy residue generated during the production of cheese and yogurt. Whey contains mainly lactose and proteins, contributing to its high chemical oxygen demand (COD). Current environmental regulations request proper whey disposal to avoid environmental pollution. Whey components can be transformed by yeast into ethanol and biomolecules with aroma and flavor properties, for example, 2-phenyethanol (2PE), highly appreciated in the industry due to its organoleptic and biocidal properties. The present study aimed to valorize agri-food residues in 2PE by developing suitable bioprocess. Cheese whey was used as substrate source, whereas crab headshells, residual soy cake, and brewer’s spent yeast (BSY) were used as renewable nitrogen sources for the yeasts *Kluyveromyces marxianus* and *Debaryomyces hansenii*. The BSYs promoted the growth of both yeasts and the production of 2PE in flask fermentation. The bioprocess scale-up to 2 L bioreactor allowed for obtaining a 2PE productivity of 0.04 g_2PE_/L·h, twofold better productivity results compared to the literature. The bioprocess can save a treatment unit because the whey COD decreased under the detection limit of the analytical method, which is lower than environmental requirements. In this way, the bioprocess prevents environmental contamination and contributes to the circular economy of the dairy industry.

## 1. Introduction

The agri-food industry is an important social and economic sector of Canada, generating 2.5 billion CAD per year. For example, this country is one of the top ten major cheese producers in the world [1,2]. The agri-food sector in Quebec is recognized for its production of cheese, seafood, cereals, legumes, and brewed beverages [3,4]. For example, the province of Quebec produces 2.49 × 10^5^ tons of cheese per year, representing 51% of Canadian production. This generates 83,000 direct and indirect jobs and shows the importance of the dairy industry in Quebec’s economy, especially in local regions [5,6].

The residues of the agri-food sector can be a big problem because they are issued in great volume and represent a huge pollutant load for environmental systems, and their management needs considerable human and material resources. Among the current managing practices for some of them, land spreading is a common practice, but it can cause soil and water pollution due to the high pollutant load [7,8]. For example, whey is the liquid residue from cheese production, and it contains about 55% of milk solids, such as lactose (from 33.6 to 52.0 g/L), proteins (from 1.8 to 10.0 g/L), and minerals (from 0.4 to 1.6 g/L). The content of solids in whey is equivalent to a chemical oxygen demand (COD) in the range from 60 to 80 g O_2_/L [9,10].

In this context, the valorization of agri-food residues is an emerging practice. The biological way to valorize them is an interesting option that decreases the pollutant load by obtaining high added-value biomolecules. New environmental rules oriented towards deviating organic residues from landfills promote the research and development of new valorization bioprocesses. Fermentation using nontraditional yeasts, such as *K. marxianus* and *D. hansenii*, is an option for agri-food management that offers several advantages because added-value biomolecules are obtained. These yeasts can hydrolyze lactose to produce ethanol and organic acids. Under specific nitrogen conditions, they can transform amino acids into fusel alcohols through the Ehrlich pathway, for example, L-Phenylalanine (L-Phe) into 2-phenylethanol (2PE) [11,12]. 2PE is highly appreciated by the pharmaceutic, food, and cosmetic industries because of its aroma, flavor, and antiseptic properties with a market price of naturally produced 2PE of around 640 USD/kg [13]. However, the 2PE production using biological methods presents several challenges to being exploited. For example, alcohols and organic acids can accumulate in the culture broth during fermentation and inhibit yeast growth. In addition, compared with chemical methods, fermentation is a time-consuming process. In order to overcome some of the limitations for whey fermentation, strategies such as cell immobilization [10,14], yeast co-culture [11], and co-substrate fermentation [15] have been explored. For example, Valdez-Castillo et al. (2021) showed that whey fermentation using *K. marxianus* and *D. hansenii* under co-culture mode led to a doubling in 2PE productivity in comparison to monocultures.

The main aim of this study was to develop a fermentation process to simultaneously treat and valorize agri-food residues into 2PE. The effect of three nitrogen-rich agri-food residues on yeast growth and 2PE production was studied, with the purpose of replacing costly raw materials. This could improve the cost–benefit of whey fermentation and contribute to developing a sustainable bioprocess for valorizing agri-food residues under the principles of the circular economy.

## 2. Results

### 2.1. Characterization of Agri-Food Residues

The chemical characterization of cheese whey, crab shells, residual soy cake, and BSY is shown in Table 1. **Cheese whey** had a pH of 4.88 ± 0.02, lactose concentration of 35.44 ± 0.00 g/kg_residue_, and methionine and leucine were the most abundant amino acids with 12.00 ± 0.00 and 5.02 ± 0.01 g/kg_residue_, respectively. All the aforementioned components contributed to a COD of 64.94 ± 2.93 g O_2_/L. **Crab headshell** was the residue with the highest content of total nitrogen and total protein, 2.18 ± 0.01 and 180.21 ± 6.61 g/kg_residue_, respectively. It also presented the highest content of cations. **Residual soy cake** had total nitrogen and total protein contents of 9.30 ± 0.04 and 85.60 ± 5.85 g/kg_residue_. The most abundant amino acid in the residual soy cake was L-Phe, with a concentration of 5.00 ± 0.10 g/kg_residue_. **Brewer’s spent yeast** (BSY) had total nitrogen and total protein contents of 13.12 ± 1.41 and 72.65 ± 0.99 g/kg_residue_, respectively. It presented the highest concentration of L-Phe (11.01 ± 0.02 g/kg_residue_) in comparison to the other residues. However, this was not enough to be considered as an L-Phe source for 2PE production.

### 2.2. Yeast Growth

**Figure 1:**
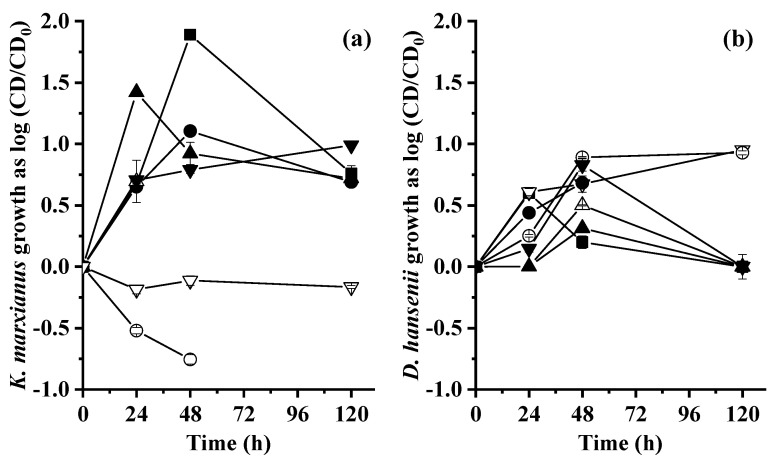
Growth of (**a**) *Kluyveromyces marxianus* and (**b**) *Debaryomyces hansenii* as co-culture mode for aerobic whey medium fermentation. Symbols indicate the co-culture growth for the ◼ whey medium control, ▼ whey medium supplemented with nonhydrolyzed crab, ▽ whey medium supplemented with hydrolyzed crab, ⚫ whey medium supplemented with nonhydrolyzed residual soy cake, ⚪ whey medium supplemented with hydrolyzed residual soy cake, ▲ whey medium supplemented with nonhydrolyzed brewer’s spent yeast, and △ whey medium supplemented with hydrolyzed brewer’s spent yeast.

Figure 1a,b shows the growth of *K. marxianus* and *D. hansenii*. Since the initial cell density ratio of each yeast was different, a normalized cell density (CD) was calculated to follow the evolution of CD for each yeast. The normalized CD was calculated using the common logarithm of the ratio of CD at any time of fermentation by the initial CD (CD_initial_) of *K. marxianus* (5.0 × 10^7^ CFU/mL) and *D. hansenii* (1.0 × 10^7^ CFU/mL). The normalized CD led to the determination of the number of times that the CD_initial_ increased over time. A positive value of normalized CD means that CD_initial_ increased, zero corresponds to no increase, and a negative value to a decrease.

For *K. marxianus* in WM, the maximum normalized CD (1.89 ± 0.01 log CD*_K. marxianus_*/CD_initial_) was observed at 48 h. This was followed by WNBSY fermentation with 1.42 ± 0.00 log CD*_K. marxianus_*/CD_initial_ at 24 h. These values corresponded to CD_initial_ of 3.85 ± 0.01 × 10^9^ and 1.31 ± 0.00 × 10^9^ CFU/mL, respectively. For WNC, a growth up to 0.99 ± 0.01 log CD*_K. marxianus_*/CD_initial_ (4.85 ± 0.15 × 10^8^ CFU/mL) at 120 h. For media with hydrolyzed residues, Figure 1 shows the *K. marxianus* growth inhibition.

**Figure 2:**
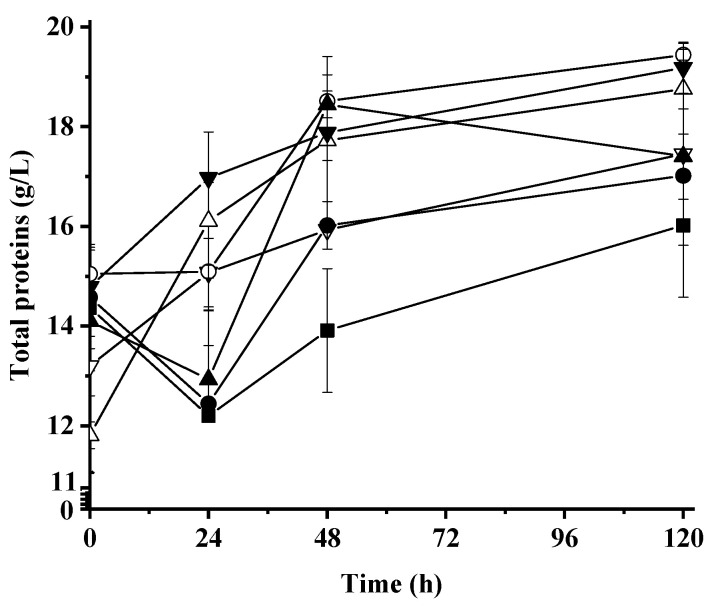
Production of total protein during aerobic whey medium fermentation using a co-culture of yeast. Symbols indicate the fermentation of ◼ whey medium control, ▼ whey medium supplemented with nonhydrolyzed crab, ▽ whey medium supplemented with hydrolyzed crab, ⚫ whey medium supplemented with nonhydrolyzed residual soy cake, ⚪ whey medium supplemented with hydrolyzed residual soy cake, ▲ whey medium supplemented with nonhydrolyzed brewer’s spent yeast, and △ whey medium supplemented with hydrolyzed brewer’s spent yeast.

Figure 2 shows the total protein concentration in culture broth for all assays. For WNC, the total protein content increased from 14.79 ± 0.79 to 17.45 ± 0.91 during fermentation, whereas for WM, WSH, and WNBSY, the total protein content decreased during the first 24 h. After this time, total protein increased up to 120 h. For media with hydrolyzed residues (WHC, WHS, and WBSY), the content of total protein always increased during the fermentation.

### 2.3. Effect of Addition of Biosourced Protein on Lactose Consumption and Ethanol Production

**Figure 3:**
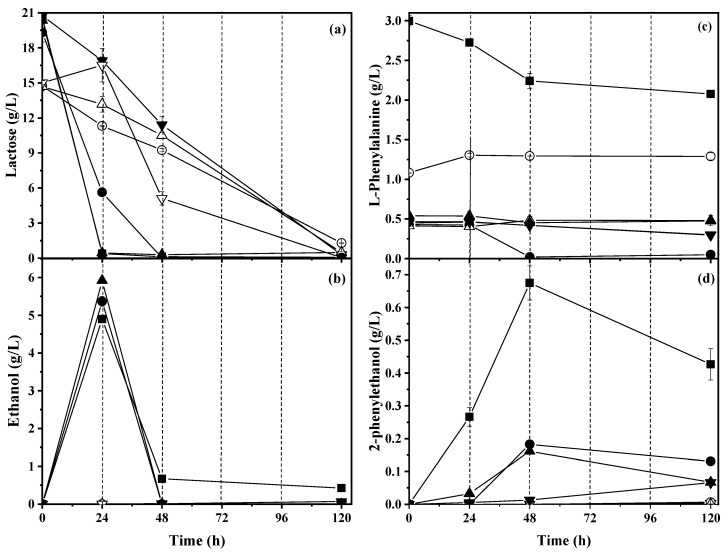
(**a**) Lactose consumption; (**b**) ethanol production; (**c**) L-phenylalanine consumption; and (**d**) 2-phenylethanol production during aerobic whey medium fermentation using a co-culture of yeast. Symbols indicate the fermentation of ◼ whey medium control, ▼ whey medium supplemented with nonhydrolyzed crab, ▽ whey medium supplemented with hydrolyzed crab, ⚫ whey medium supplemented with nonhydrolyzed residual soy cake, ⚪ whey medium supplemented with hydrolyzed residual soy cake, ▲ whey medium supplemented with nonhydrolyzed brewer’s spent yeast, and △ whey medium supplemented with hydrolyzed brewer’s spent yeast.

Figure 3a shows the lactose concentration during the fermentation assays. For the media with no hydrolyzed residues, the initial average lactose concentration was 20.24 ± 0.55 g/L. For WM and WBSY, the lactose had been depleted at 24 h. For WNC and the media with hydrolyzed residues, the initial average lactose concentration was 14.81 ± 0.14 g/L, and consumption of lactose took more time, being completely depleted at 120 h.

Figure 3b shows the ethanol concentration during the fermentation. For WNBSY, WM, and WNS, their highest ethanol concentration was observed at 24 h and then it decreased up to complete consumption. The highest ethanol concentration was observed for WNBSY, 5.92 ± 0.05 g/L, for a productivity of 0.30 ± 0.01 g_ethanol_/L·h. In the case of WNC and media with hydrolyzed residues, ethanol production was completely inhibited during fermentation.

Table 2 shows the rates of consumption of lactose and L-Phe, and productivity of alcohols. For WM and WBSY, the average lactose consumption rate was 0.83 ± 0.03 g_lactose_/L·h, whereas for WNC and media with hydrolyzed residues, it ranged from 0.11 to 0.17 g_lactose_/L·h.

### 2.4. Effect of Addition of Biosourced Protein on L-Phenylalanine Consumption and 2-Phenylethanol Production

Figure 3c shows the concentration of L-Phe during the fermentation assays. Since the residues used to formulate each media contained proteins, the initial concentration of L-Phe was different for each one. WM had the highest initial L-Phe concentration of 3.0 ± 0.1 g/L because it was enriched with pure L-Phe. The initial L-Phe concentration for the other media ranged from 0.5 to 1.0 g/L.

For WM, the highest L-Phe consumption rate (15.75 ± 0.65 mgL-Phe/L·h) was observed and for the rest of the media with nonhydrolyzed and hydrolyzed residues, the L-Phe consumption rate ranged from 0.40 to 8.55 mgL-Phe/L·h.

Figure 3d shows the 2PE concentration during the fermentation assays. The highest 2PE concentration was 674.9 ± 52.1 mg/L at 48 h in WM. WNS and WNBSY had 182.3 ± 0.0 and 161.3 ± 1.2 mg 2PE/L, respectively, at 48 h of fermentation. For the three media, the 2PE concentration decreased at 120 h. The highest 2PE yield (2.44 ± 0.10 mg 2PE/mgL-Phe) was observed for WNBSY. For the media with hydrolyzed residues, 2PE production was inhibited.

### 2.5. Co-Fermentation of Whey and Brewer’s Spent Yeast in Aerated Bioreactor

#### 2.5.1. Yeast Growth and Protein Production under Controlled Aeration

Table 3 shows the initial conditions of fermentation and calculated kinetics parameters for WM and WBSY fermentation performed in 2 L bioreactors. For WM, the initial lactose concentration was 19.7 ± 0.3 g/L. The nitrogen source was peptone and yeast extract, leading to an initial total nitrogen concentration of 3.6 ± 0.3 g/L. This corresponds to a C/N ratio of 4.9 ± 0.3 g/g. For WBSY, the initial lactose concentration was 19.6 ± 0.1 g/L. The nitrogen source was BSY, leading to an initial total nitrogen concentration of 2.2 ± 0.1 g/L and a C/N ratio of 5.5 ± 0.1 g/g.

**Figure 4:**
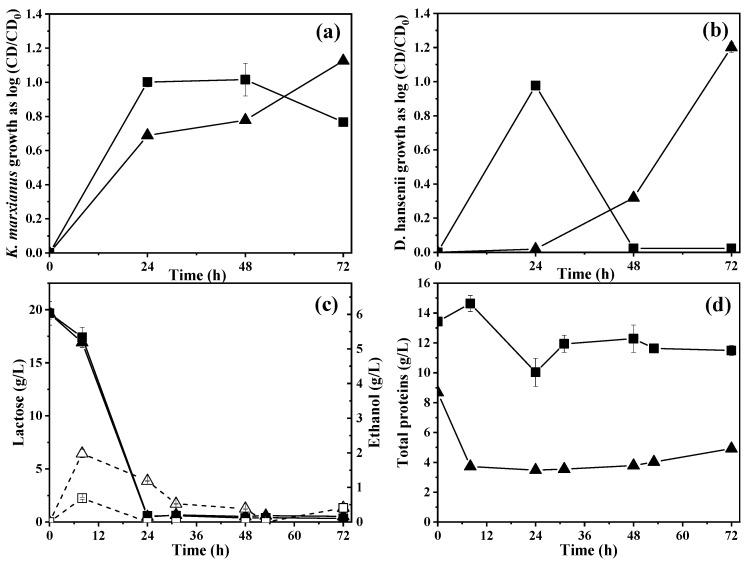
Kinetics of WM and WBSY fermentation. Growth of (**a**) *Kluyveromyces marxianus* and (**b**) *Debaryomyces hansenii* as co-culture mode for aerobic whey medium fermentation in bioreactors. (**c**) Total protein and (**d**) lactose consumption and ethanol production during the aerobic whey medium fermentation in bioreactors. Closed symbols indicate the growth of yeast, lactose consumption, and total protein concentration for the fermentation of ◼ WM control and ▲ WBSY. Open symbols indicate the ethanol production for the fermentation of □ WM-control and △ WBSY.

Figure 4a shows the growth of *K. marxianus* expressed as the normalized CD. The initial cell density for *K. marxianus* was 5 × 10^7^ CFU/mL. For WM and WBSY, the respective normalized CD was 1.00 ± 0.03 and 0.69 ± 0.00 log CD*_K. marxianus_*/CD_initial_ at 24 h. For WM, *K. marxianus* presented its stationary growth phase from 24 to 48 h and then the CD declined, whereas for WBSY, the yeast continued to grow without presenting a stationary phase.

For WM and WBSY fermentation in the bioreactor, the normalized CD*_K. marxianus_* was 1.0 ± 0.1 and 0.8 ± 0.0 log CD*_K. marxianus_*/CD_initial_ at 48 h, whereas in flask fermentation, the normalized CD was 1.8 and 1.2 higher than the normalized CD in bioreactors. This could be attributed to the shear stress on cells generated by agitation and controlled aeration.

Figure 4b shows the growth of *D. hansenii* expressed as the normalized CD. The initial normalized CD was 1 × 10^7^ CFU/mL. For WM, a cell density of 1.0 ± 0.0 log CD*_D. hansenii_*/CD_initial_ (9.0 ± 0.5 × 10^7^ CFU*_D. hansenii_*/mL) was observed. For WBSY, the yeast presented a lag phase from 0 to 48 h. The yeast had the highest normalized CD of 1.2 ± 0.0 log CD*_D. hansenii_*/CD_initial_. (1.6 ± 0.1 × 10^8^ CFU*_D. hansenii_*/mL) at 72 h.

For WM, the growth of the *D. hansenii* was similar to flask fermentation, whereas for WBSY, the long lag phase could be attributed to shear stress as it was observed for *K. marxianus*.

Figure 4c shows the total protein concentration for WM and WBSY. For WM, a decrease from 13.4 ± 0.2 to 6.0 ± 0.9 g/L at 24 h was observed; then, the protein content increased and stayed around 11.9 ± 0.6 g/L from 31 to 72 h. For WBSY, total protein decreased from 8.7 ± 0.1 to 3.7 ± 0.0 g/L at 8 h, and it was nearly constant until the end of fermentation.

#### 2.5.2. Effect of Controlled Aeration on Lactose Consumption and Ethanol Production

Figure 4d shows the consumption of lactose and the production of ethanol. For WM and WSBY, lactose was completely depleted before 24 h. For WBSY, the highest ethanol concentration (2.0 ± 0.1 g/L) was observed at 8 h, corresponding to a yield of 0.7 ± 0.0 g_ethanol_/g_lactose_ because of the low lactose consumption at this time. For WBSY, the ethanol was consumed slower than in WM.

#### 2.5.3. Effect of Controlled Aeration on the Consumption of L-Phenylalanine and Production of 2-Phenylethanol

**Figure 5:**
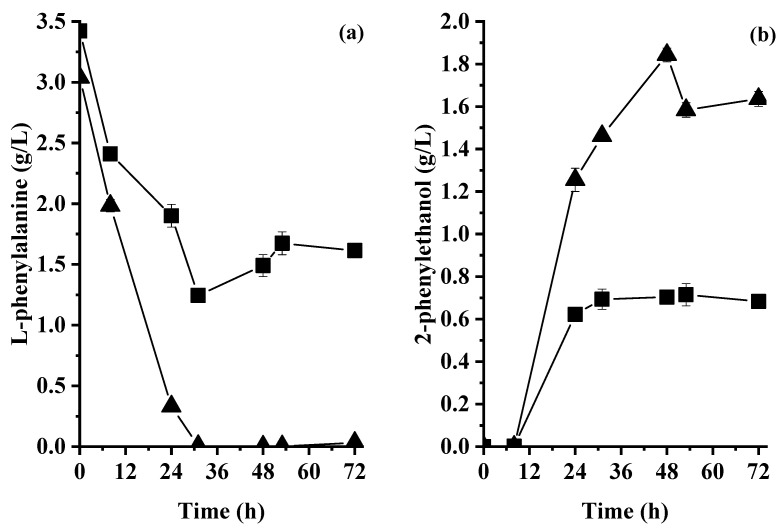
(**a**) L-phenylalanine concentration consumption and (**b**) 2-phenylethanol production during the aerobic whey medium fermentation in bioreactors. Symbols indicate co-culture performance for the fermentation of ◼ WM control and ▲ WBSY.

Figure 5a shows the L-Phe concentration in the culture broth during fermentation in bioreactors. For WM, the concentration of L-Phe decreased from 3.4 to 1.2 g/L, showing an apparent L-Phe consumption rate of 0.06 ± 0.00 g_L-Phe_/L·h at 31 h, whereas for WBSY, the L-Phe was depleted at 31 h, corresponding to an apparent L-Phe consumption rate of 0.1 ± 0.0 g_L-Phe_/L·h.

Figure 5b shows the concentration of 2PE during the fermentation of WM and WBSY. For WM, the highest 2PE concentration (0.69 ± 0.09 g/L) was observed at 31 h, corresponding to a productivity and a yield of 0.02 ± 0.00 g_2PE_/L·h and 0.31 ± 0.00 g_2PE_/g_L-Phe_, respectively. For WBSY, the highest 2PE concentration (1.84 ± 0.03 g/L) was observed at 48 h, corresponding to a productivity of 0.04 ± 0.00 g_2PE_/L·h and a yield of 0.61 ± 0.01 g_2PE_/g_L-Phe_. For WBSY, 2PE productivity was 2.35-fold higher than the 2PE productivity observed for WM fermentation. In addition, 2PE productivity for WBSY was 12.12-fold higher than that observed for WNBSY fermentation in a flask.

## 3. Discussion

### 3.1. Characterization of Agri-Food Residues

The lactose content of the cheese whey used was in the range of lactose content reported for acid whey from 36 to 52 g/L [16,17]. For protein content, rennet is a protease used to break milk proteins and form curd, affecting the final concentration of proteins of cheese whey [16,17,18]. The major protein fraction in acid whey is α-lactalbumin, β-lactoglobulin, and bovine serum albumin, which are rich in methionine, leucine, and isoleucine [18]. This is in accordance with the abundance of amino acids observed for whey (Table 1).

For **crab headshells**, the high content of total nitrogen can be attributed to the presence of crab meat attached to the shell, and also to the content of proteins and chitin in the shell itself. Chitin is a long-chain polymer of N-acetylglucosamine. The total protein concentration could be related to the species, gender, and capture season. It can also be dependent on the crab shell part analyzed (head, thorax, or abdomen). Pires et al. (2017) [19] studied the chemical composition of four crab species based on their gender and the year and season when the crab was captured. They observed a total protein content from 80 to 210 g/kg. The protein content of 180.21 ± 6.61 g/kg for crab headshells was in accordance with Pires et al.’s observations, being in the high limit of the range.

The high concentration of cations in the crab headshells was mainly caused by the CaCO_3_ content and the complexes between K^+^ and Mg^2+^ with phosphorous, sulfur, and chlorine, affording hardness to the crab exoskeletons. The content of Ca^2+^ for crab has been reported in the range from 169 to 519.5 g/kg, for K^+^ from 1.9 to 2.8 g/kg, and for Mg^2+^ from 10.8 to 26.9 g/kg [19,20]. The values of the present study comprise these ranges (Table 1).

For **residual soy cake**, the total nitrogen and total protein content can be attributed to two main proteins called conglycinin and glycinin, which are glycoproteins rich in cysteine and glycine [21,22]. Stanojevic et al. (2013) [21] studied six varieties of soybean to produce soy milk and residual soy cake. They observed an average of total nitrogen and protein content of 57.46 ± 4.24 and 359.13 ± 26.51 g/kg_residue_, respectively. These values are 6.17- and 4.19-fold higher than the total nitrogen and protein contents determined for the residual soy cake used in the present study. These differences can be attributed to the handling of soybean processing.

For **BSY**, the high concentration of total nitrogen and total protein could be related to the processes for cell debittering, lysis, and storing performed during beer brewing and management of residue. The concentration of total protein is also highly related to the yeast strain and chemical composition of organelles, membranes, and cell walls. For example, the cell wall contains chitin and glycoproteins, the latter are composed of proteins and polysaccharides. The average content of proteins reported in the cell wall was 39.90 ± 3.52 g/kg_residue_ (dry basis) [23,24], whereas the total protein of the whole BSY cells ranged from 480 to 730 g/kg_residue_ (dry basis) [25,26].

### 3.2. Yeast Growth

The slow growth of *K. marxianus* in media with nonhydrolyzed residues could be attributed to the high concentration of cations released from residues during their hydrolysis. For example, Mg^2+^ is a macronutrient used as a co-factor for the proteins of the cell membrane of yeasts. These proteins regulate the mass transport from the media to the cytosol of yeasts [27,28]. The high content of Ca^2+^ in crab headshells could mimic the co-factor action of Mg^2+^, decreasing the *K. marxianus* growth in WNC. The hydrolysis of residues and their neutralization added Cl^−^ and increased the concentration of K^+^. The concentration of both Cl^−^ and K^+^ in the hydrolyzed media was calculated considering the volume and the molarity of either HCl or KOH. For example, for WHC, WHS, and WHBSY media, 10 mL of HCl (6M) was used to hydrolyze the residues. The hydrolyzed solution was neutralized with 7.5 mL of KOH (6M). This produced hydrolyzed and neutralized media with concentration of 70.98 and 58.69 g/L of Cl^−^ and K^+^, respectively. The high concentration of ions for these media could inhibit yeast growth by osmotic stress, which affects the water retention capacity of cells and inhibits the oxidation of adenosine triphosphate, as indicated by Illarionov et al. (2021) [28] when they studied the effect of the initial concentration of Cl^−^ and K^+^ on the growth of *K. marxianus* during glucose fermentation. They observed this phenomenon with complete inhibition at an initial concentration of 70.80 and 78.20 g/L of Cl^−^ and K^+^, respectively.

*D. hansenii* adapted better than *K. marxianus* for the fermentation of hydrolyzed residues, allowing for the predominance of *D. hansenii*. The maximum 2.1 ± 0.1 log CD*_D. hansenii_*/CD_initial_ was observed at 48 h for WHS fermentation and 120 h for WHC fermentation.

*D. hansenii* is a halotolerant yeast that is resistant to stressful osmotic environments. Under osmotic stress, *D. hansenii* can produce and accumulate glycerol to create an osmotic pressure balance between the environment and cytosol [29]. This could allow for better adaptation to the high concentration of ions in media with hydrolyzed residues. Calahorra et al. (2009) [29] studied the effect of KCl (up to 3.9 g/L) and NaCl (up to 2.3 g/L) on *D. hansenii* during glucose fermentation, observing that there is a correlation between salt concentration and glycerol production.

For the media with nonhydrolyzed residues, yeast extract and peptone were the nitrogen source and the decrease in protein content within 24 h of fermentation could be due to the quick metabolization of soluble protein from these sources. From 48 to 120 h, the increase in total protein concentration could be produced by the lysis of yeast cells during the stationary growth phase and the death phase. The hydrolysis of agri-food residues rich in protein, such as glycoproteins, could contribute to an increase in total protein concentration in the media [30,31]. For example, Foukis et al. (2012) [31] cultivated *K. marxianus* in glucose base medium containing 0.4 g/L of bovine serum albumin. They identified a protease that broke the amino acids from the carboxyl end of the proteins in bovine serum albumin. In addition, Kumura et al. (2002) [30] studied the fermentation of synthetic milk ultrafiltrate containing casein at pH 6.2 and 20 °C using *D. hansenii*. They observed the degradation of α- and β-casein when the intracellular proteins of the yeast were extracted.

In the case of hydrolyzed residues, the continuous increment in total protein concentration could be associated with protein synthesis by yeast and their accumulation in the media.

### 3.3. Effect of Addition of Biosourced Protein on Lactose Consumption and Ethanol Production

According to the glycolysis pathway, lactose was hydrolyzed by the yeasts and its oxidation led to the formation of pyruvate and energetic molecules such as adenosine triphosphate (ATP) and nicotinamide adenine dinucleotide hydrogen (NADH). Then, pyruvate was further oxidized, leading to its use in biomass and biomolecule synthesis [10,32]. Lactose consumption of WNC and hydrolyzed residues was slow because of partial or complete inhibition of growth, as shown in Figure 1. Lactose consumption depends on its transportation from media to cytosol by lactose permease and the proton motive force in the cell wall, this being an energy-consuming process. The diffusion and accumulation of ions such as K^+^, Na^+^, and Cl^−^ in the vacuoles and cytosol of yeasts could decrease the energy obtained from the activity of H^+^-ATPase coupled with the hydrolysis of ATP to ADP and phosphate and the proton motive force, limiting lactose transport [28,33].

Ethanol is produced by the pyruvate oxidation pathway. Pyruvate is oxidized into acetaldehyde and then further oxidized to produce ethanol as the end product. When lactose was depleted at 24 h of fermentation for WM, WNBSY, and WNS, the yeast could change their metabolism to oxidize ethanol and then use it as carbon source. This caused a decrease in ethanol concentration after 24 h. This observation was in accordance with previous studies performed by Valdez Castillo et al. (2021) [11]. They observed that whey fermentation using *K. marxinus* and *D. hansenii* under co-culture mode produced ethanol during the first 24 h, completely consuming the lactose; then, ethanol was consumed.

### 3.4. Effect of Addition of Biosourced Proteins on L-Phenylalanine Consumption and 2-Phenylethanol Production

For WNC, WNS, and WNBSY, the initial L-Phe concentration is attributed to the yeast extract and peptone that were added to the media, whereas the low initial L-Phe concentration in the media with the hydrolyzed residues could be attributed to the degradation of amino acids during hydrolysis with HCl.

The consumption of L-Phe is regulated by its initial concentration and its transamination to obtain energy during the exponential growth phase [34,35]. L-Phe transamination depends on the presence of α-ketoglutarate, which is the electron acceptor of the reaction. However, α-ketoglutarate is also an intermediate of the tricarboxylic acid cycle used to produce energetic biomolecules for cell growth [12]. In the present study, when the carbon source was completely depleted in the media, the α-ketoglutarate could mainly be used in L-Phe transamination instead of the tricarboxylic acid cycle to produce energy for cell preservation.

L-Phe can be consumed and transformed via the Ehrlich pathway into 2PE. According to this pathway, 2PE production is an energy-consuming process [12]. According to this, when yeast depleted the lactose, the production of 2PE could be limited, and the energy obtained from ethanol and L-Phe consumption could be deviated to the preservation of cells. The high yield of 2PE for the WNBSY could be attributed to the continuous hydrolysis of BSY residual yeast cells during fermentation. This allowed for a constant L-Phe concentration in the medium despite its consumption and transformation.

The statistical analysis shows that the use of the nonhydrolyzed residual soy cake and BSY had a positive effect on 2PE production in comparison to the other nonhydrolyzed and hydrolyzed residues (Supplementary Material Table S1). Nonhydrolyzed BSY was selected as a nitrogen source for the second step of this study because it promoted the growth of the yeasts and maintained the concentration of L-Phe and the production of 2PE.

### 3.5. Co-Fermentation of Whey and Brewer’s Spent Yeast in Aerated Bioreactor

#### 3.5.1. Yeast Growth and Protein Production under Controlled Aeration

WM was enriched with yeast extract and peptone, which contain highly digestible peptides that could be quickly consumed at 24 h. From 31 to 72 h, the observed total protein could be attributed to the stationary phase of both *K. marxianus* and *D. hansenii*. During this period, a balance between new cells and the death of cells is expected, allowing for the constant normalized CD previously described. In this context, the dead cells could release proteins to the media, increasing the total protein concentration. In the case of WBSY, the residual cells of brewer yeast were the source of nitrogen. The hydrolysis of components of dead cells, such as glycoproteins from the cell wall could release proteins [23,25]. The soluble proteins in WBSY could be easily consumed from 0 to 8 h, allowing for a decrease in total protein concentration and facilitating the growth of both *K. marxianus* and *D. hansenii*.

#### 3.5.2. Effect of Controlled Aeration on Lactose Consumption and Ethanol Production

The airflow rate of 1 L/min allowed for maintaining a level of 30% of oxygen saturation in both culture media. Oxygen is the last electron acceptor of the electron chain under aerobic fermentation of lactose. This means that the oxidation of lactose occurred first, and ethanol later released carbon and energetic biomolecules for the yeast metabolism, for example, biomass synthesis. In comparison with flask fermentation, the aeration increased the activity of yeasts, which quickly consumed the carbon sources, limiting ethanol accumulation. Similar results were observed by Beniwal et al. (2017) [32] when they studied the aerobic fermentation of whey using *K. marxianus* in a 3 L bioreactor, observing an ethanol concentration of 2 g/L at 8 h of fermentation.

Mg^2+^ is a co-factor for ATPase and permeases (membrane transport proteins) in the plasma membrane, regulating the proton motive force. In this context, Mg^2+^ could promote the transport of the ethanol produced from the cytosol to the culture media [27,28]. Once the ethanol was accumulated in the media at 8 h, it could act as a toxic compound for yeast cell membranes. Thus, the Mg^2+^ could act as a protective agent, delaying the consumption of ethanol and preventing yeast membrane damage during WBSY fermentation (Supplementary Material Table S3). Deesuth et al., 2015 [27], studied the fermentation of sweet sorghum juice (200 g/L of total sugars) using *Saccharomyces cerevisiae* for the production of ethanol. The medium was fermented with and without dried BSY (13.5 g/L), and the production of ethanol was higher using BSY. This was attributed to the content of Mg^2+^ (50 mg/L) in BSY.

The initial soluble COD of WM and WBSY was 26.0 ± 0.5 and 25.1 ± 0.2 g O_2_/L, respectively (Supplementary Material Table S3). The final soluble COD was under the limits of detection for both culture broths. According to the Canadian regulatory limits of discharge of wastewater system effluents regulation SOR/2012-139, the final soluble COD content in media fits this limit of discharge (≤0.02 g O_2_/L) and no supplementary treatment is needed.

#### 3.5.3. Effect of Controlled Aeration on the Consumption of L-Phenylalanine and Production of 2-Phenylethanol

For WM and WBSY, the L-Phe consumption under controlled aeration conditions was 2.2 ± 0.0 and 3.0 ± 0.0 g_L-Phe_/L, respectively. This is 2.9 and 30.7 times higher than the L-Phe consumed in the flasks for WM and WNBSY, respectively. These differences could be associated with the high activity of yeasts caused by aeration. The controlled aerated environment contributed to the oxidation of the carbon source and then of other nutrients. Authors of [36] studied the effect of three nitrogen sources on the consumption of sugars and growth of four yeast strains under controlled and noncontrolled aeration conditions. They observed that peptone enhanced the growth rate of the yeast strains in comparison with casamino acids and ammonium sulfate. They also observed faster nutrient consumption when yeasts were incubated under controlled aeration conditions. This is in agreement with the results observed in the present study.

For flask fermentation, the production of energetic molecules needed for 2PE production could be limited by several factors, including aeration. In the case of bioreactors, aeration was not a limiting factor, and yeasts could transform L-Phe into 2PE faster than by fermentation in flasks. Statistical analysis showed that 2PE production was facilitated when BSY was used as a supplement of whey in comparison to the use of yeast extract and peptone (Supplementary Material Table S2). Alonso-Vargas et al. (2022) [34] studied the fermentation of whey enriched with 4.5 g/L of L-Phe and 1 g/L of (NH_4_)_2_SO_4_ using *K. marxianus*. They observed a 2PE concentration of 1.17 g/L at 72 h, leading to a productivity of 0.02 g_2PE_/L·h. According to our knowledge, this is the best performance reported for *K. marxianus* used to produce 2PE. The 2PE concentration and productivity observed in the present study using co-cultured yeast to ferment whey and BSY are 1.5- and 2.0-fold higher than the values reported by Alonso-Vargas et al. (2022) [34]. In addition, the lactose and nitrogen are from wastes, showing the potential of biosourced raw materials to produce high value-added molecules.

Regarding the replacement of commercially available compounds used for preparing the culture medium, such as yeast extract and peptone by an agri-food residue (BSY), an economic advantage is observed when comparing the costs of both culture media and the production of high value-added byproducts. This comparison is shown in the Supplementary Material and Table S4. The economic comparison shows that in a basis production of 1000 L of culture medium, the use of commercially available ingredients to prepare it represents a cost of 658.25 USD/1000 L, whereas the cost for a culture medium using the residue was 118.25 USD/1000 L. This corresponds to saving of 82% when BSY is used for fermentation.

Considering the current market price for 2PE naturally produced of 640 USD/kg, the investment of raw material cost for fermentation using commercial ingredients cannot be recovered because the mass of 2PE produced in this volume of culture medium is 0.7 kg, whereas the use of BSY led to obtaining 1.8 kg for the same volume, representing an income of 1152 USD from an investment of 118 USD. For this case, the difference between investment and income allowed for the coverage of transport fees and energy consumption for whey fermentation, considering a scenario of local production. This confirms the potential of the bioprocess using cheese whey and BSY for yeast fermentation, and its feasibility must be verified by a sale-up to obtain more technical data that will allow for accurate calculation in a detailed economic study.

## 4. Materials and Methods

This study was performed in two steps: The first step aimed to ferment cheese whey using a co-substrate (crab headshells, residual soy cake, and brewer’s spent yeast) as a biobased protein source to produce 2PE. The second step aimed to scale up the fermentation of best combination whey–co-substrate in bioreactors of 2 L and to study the effect of aeration on the production of 2PE.

### 4.1. Agri-Food Residues: Handling and Characterization

Cheese whey, crab headshells, residual soy cake, and brewer’s spent yeast (BSY) were the substrates used for fermentation assays. Residues were obtained from Canadian agri-food enterprises (Quebec). Cheese whey was conserved at −20 °C until its use. The crab headshells were rinsed with distilled water, dried at 60 °C for 48 h (Thermo Herathern Fisher oven, OMS180, USA), and submitted to a grinding mill (Fritsch Universal Cutting Mill 19.5720/021704, Germany) to obtain particles with sizes from 1 to 2 mm. These particles were milled with a pestle and mortar into fine powder. The residual soy cake was dried at 60 °C for 48 h (Thermo Herathern Fisher oven, OMS180) and milled with a pestle and mortar into fine powder. The BSY was conserved at −20 °C until being used for fermentation assays.

The residues were characterized to determine the content of total nitrogen (Kjeldahl analyzer, Foss, Kjeltec 8200, Denmark), total solids, ashes [37], total protein (Lowry’s method), and cations (inductively coupled plasma–optical emission spectroscopy, ICP-OES OPTIMA 4300 DV, Australia). For cheese whey and BSY, the pH (Fisher Scientific benchtop pH meter, Accumet AB250, Singapore), content of COD (HACH kit and spectrophotometer, Canada), and dissolved organic carbon (TOC-L analyzer, Shimadzu, Canada) were determined according to standard methods [37].

### 4.2. Culture Media

LYP medium was used for preparing the inoculum. It contained lactose 45 g/L (Spectrum chemical MFG corp, CA, USA >98%), yeast extract 10 g/L (Organotechnie S.A.S, Terrebonne, QC, Canada), and peptone 20 g/L (Organotechnie S.A.S, Terrebone, QC Canada). A whey-based medium was used for fermentation assays. It was prepared by diluting the cheese whey with distilled water to obtain a lactose concentration of 20 g/L. Yeast extract 10 g/L and peptone 20 g/L were added.

For the first step of the study, the whey-based medium was enriched with L-Phe for a concentration of 3 g/L (BioShop life science products, Burlington, ON, Canada, >98%) (WM). Nonhydrolyzed and hydrolyzed crab headshells 0.9 g, residual soy cake 2.0 g, and BSY 0.9 g were added to each flask to enrich the whey-based medium for an initial total protein concentration of 14.7 ± 2.0 g/L for all conditions. Whey-based media with nonhydrolyzed crab headshells, residual soy cake, and BSY were identified as WNC, WNS, and WNBSY, respectively, whereas the whey-based media with hydrolyzed crab headshells, residual soy cake, and BSY were identified as WHC, WHS, and WHBSY, respectively. The residues were hydrolyzed as described in Section 4.7. The pH of each medium was adjusted to 6.5 with KOH 6M (Sigma-Aldrich, Mississauga, ON, Canada, >99%) or HCl 6M (Fisher, Ottawa, ON, Canada, 37% *v/v*). Then, 30 mL of media were placed in Erlenmeyer flasks of 250 mL and sterilized using an autoclave at 120 °C for 15 min.

### 4.3. Inoculum

*Kluyveromyces marxianus* NRRL Y-1109 and *Debaryomyces hansenii* NRRL Y-1448 were acquired from the culture collection of the United States Department of Agriculture (USDA). The yeasts were seeded separately in 50 mL of LYP in 250 mL flasks. They were incubated at 25 °C and 200 rpm for 20 h in a rotary incubator (INNOVA 44 New Brunswick Scientific). For the fermentation assays, both *K. marxianus* and *D. hansenii* were inoculated for an initial OD_600_ (UV-VIS spectrophotometer, Eppendorf BioPhotometer plus) of 0.5 and 0.1, corresponding to initial cell densities of 5.0 × 10^7^ and 1.0 × 10^7^ CFU/mL, respectively. This co-culture ratio was selected based on a previous study (Valdez Castillo et al., 2021) [11].

### 4.4. Fermentation Conditions

Fermentations were carried out at 25 °C and 200 rpm for 120 h. Samples of culture broth were taken at 0, 24, 48, and 120 h to determine cellular density, and concentration of lactose, L-Phe, ethanol, and 2PE.

Samples were handled as indicated by Valdez-Castillo et al. (2021) [11]. Briefly, 1 mL sample was centrifuged at 16,639× *g*, 4 °C for 2 min (Eppendorf 5804 R-Benchtop centrifuge). The supernatant was used to determine total protein, amino acid, lactose, ethanol, and 2PE concentrations. One hundred microliters of non-centrifuged sample was used to determine cellular density using the plate counting method, dilutions from 10^−3^ to 10^−8^ on solid LYP with 15% (*w/v*) of agar, and incubated at 25 °C for 24–48 h.

Lactose and L-Phe consumption rates were determined by considering the difference between the initial and their lowest concentration divided by the elapsed time of fermentation in which the latter was observed. Ethanol and 2PE productivities were determined by considering the differences between the initial and highest concentration divided by the elapsed time of fermentation in which the latest was observed.

### 4.5. Batch Fermentation in 2 L Bioreactor

Diluted cheese whey was enriched with 61.11 g/L of BSY and 3 g/L of L-Phe (WBSY). BSY was used as the nitrogen source instead of yeast extract and peptone. WM was used as control.

Fermentations were carried out in 2 L stirred tank bioreactors (New Brunswick scientific BioFlo/Celligen 115, USA). A quantity of 1.8 L of either WM or WBSY medium was added to the bioreactor and sterilized at 121 °C for 30 min using an autoclave (Steris Amsco Lab 250). The inoculum was added to bioreactors as mentioned before. The agitation cascade system was from 300 to 600 rpm. The pH was maintained at 6.5 ± 0.5 using HCl and KOH solutions 4M. The temperature was maintained at 25 °C and controlled with a chilling system and heating jacket. Aeration was set to maintain 30% of oxygen saturation in media using an aeration rate of 0.5 of volume of air per unit volume of medium per minute (vvm). Samples of culture broth were taken at 0, 8, 24, 31, 48, 53, and 72 h to determine the concentration of lactose, L-Phe, ethanol, 2PE, TOC. Total protein and amino acid concentrations were determined following the Lowry method and derivatization of amino acids as described below. Samples were handled as indicated in Section 4.4.

### 4.6. Determination of Protein Content Using Lowry’s Method

The extraction of proteins from solid residues (crab headshells, residual soy cake, and BSY) was performed using alkaline hydrolysis, adapting the method reported by Alabaraoye et al. (2018) [38]. One gram of each residue was placed in Erlenmeyer flasks of 250 mL and 20 mL of NaOH 1 M (Sigma-Aldrich, Ottawa, ON, Canada, >98%) was added and agitated at 250 rpm at 60 °C for 30 min. Bovine serum albumin (Sigma-Aldrich, Ottawa, ON, Canada, >98%) in distilled water was used as the standard for the calibration curve.

Protein content was determined by adapting the colorimetric Lowry’s method reported by Lucarini and Kilikian (1999) [39]. Two hundred and fifty microliters of the sample was taken and mixed with 1.25 mL of freshly prepared solution of CuSO_4_*5H_2_O 2% *w/v* (Sigma-Aldrich, St. Louis, MO, USA, >98%), Na_2_HPO_4_ 4% *w/v* (Fisher, Ottawa, ON, Canada, >98%), and Na_2_CO_3_ 3 % *w/v* (Wholesale JMC, Montreal, QC, Canada, >98%) in 0.1 M NaOH solution. This mix was homogenized by vortexing for 30 s, and it reacted for 20 min. Then, 250 µL of Folin–Ciocalteu reagent (Sigma-Aldrich, Ottawa, ON, Canada, 2M) diluted with distilled water at a ratio of 1:1 was added and homogenized by vortexing for 30 s, and it was left to react for 40 min. The absorbance was determined at a wavelength of 750 nm (DR 2700 BenchPlus spectrophotometer, HACH).

### 4.7. Quantification of Amino Acids

The extraction of amino acids from residues was performed by using acid hydrolysis. One hundred micrograms of each residue was placed in screwcap polypropylene tubes (17 mm × 120 mm) and 10 mL of 6 M HCl (Fisher, Ottawa, ON, Canada, 37% *v*/*v*) was added. The suspension was homogenized by vortexing for 30 s. The air of tubes was replaced with nitrogen, and tubes were placed in an electric oven (Thermo Herathern Fisher oven, OMS180) at 105 °C for 24 h. For culture media preparation using hydrolyzed residues, suspensions of 0.9 g of crab headshells, 2.0 g of residual soy cake, and 0.9 g of BSY in 10 mL of 6M HCl were submitted to the acid hydrolysis method as aforementioned.

Hydrolyzed samples; culture broth samples of fermentation assays; amino acid standards valine, methionine, isoleucine, leucine, and tryptophan (Sigma-Aldrich, St. Louis, MO, USA >99%); and L-Phe (BioShop life science products, Burlington, ON, Canada, >98%) were derivatized for the analysis of amino acids, adapting the method of Vilasoa-Martínez et al. (2007) [40] as follows: Methanol, triethylamine, (Fisher, Ottawa, ON, Canada, HPLC grade) and disodium hydrogen phosphate (Fisher, Ottawa, ON, Canada, >98%), and phenyl isothiocyanate (Sigma-Aldrich, Ottawa, ON, Canada, >99%) were used. Twenty microliters of the sample were placed in a vial and dried at 65 °C for 2 h. Thirty microliters of methanol–water–triethylamine (4:4:1) solution were added and vigorously mixed with a vortex for 10 s and dried at 65 °C for 10 min. Thirty microliters of methanol–water–triethylamine–phenyl isothiocyanate (7:1:1:1) were added and vortexed for 30 s. The vial was left at room temperature for 20 min and then dried for 15 min at 65 °C. Then, 150 μL of 5 mM Na_2_HPO_4_ with 5% *v/v* acetonitrile (Fisher, Ottawa, ON, Canada, > HPLC grade) solution was added to the dried sample. The pH was adjusted to 7 with 10 µL of phosphoric acid 1.07 M (Fisher, Ottawa, ON, Canada). Tubes were vortexed for 60 s. Finally, 100 μL of the neutral derivatized amino acid solution was diluted with 400 μL of 5 mM Na_2_HPO_4_ with 5% *v/v* acetonitrile. A standard solution, containing 1 mM of L-Phe in HCl 0.1 M, was obtained and derivatized as aforementioned.

The derivatized samples were analyzed using high-performance liquid chromatography (HPLC, Agilent Technologies 1260 infinity, Germany) with a UV-visible forward optical scanning detector. The HPLC was equipped with a ZORBAX Eclipse XDB-C18 (Agilent Technologies Inc., USA) column with a length of 250 mm, an internal diameter of 4.6 mm, and a film thickness of 5 µm. The temperature of the column was set at 27 °C. Two mobile phases were used. Mobile phase A was 0.14 M sodium acetate (Fisher, Ottawa, ON, Canada, >99%) buffer containing trimethylamine 0.05% *v/v* (Fisher, Ottawa, ON, Canada, HPLC grade) at a pH of 6.2, adjusted with acetic acid (Fisher, Ottawa, ON, Canada, HPLC grade). Mobile phase B was a solution of acetonitrile (Fisher, Canada, HPLC grade) and water (60:40). A gradient of the two mobile phases was used as described by Vilasoa-Martínez et al. (2007) [40]. The flow rate was set at 0.9 mL/min and the detection wavelength was 254 nm.

### 4.8. Quantification of Lactose and Ethanol

The samples were thawed to 4 °C for analysis. HPLC (Agilent Technologies 1260 infinity, Germany) coupled with a diode array detector of 1260 RID and 1290 DAD was used to quantify lactose and ethanol, respectively. Lactose (Spectrum chemical MFG corp, CA, USA >98%) and ethanol (Fisher, Ottawa, ON, Canada, > HPLC grade) were used as standards for the calibration curve. The HPLC was equipped with an Aminex HPX-87H (Agilent technologies, USA) column with a length of 300 mm, an internal diameter of 7.8 mm, and a film thickness of 5 µm. The column temperature was set at 50 °C with a mobile phase of sulfuric acid 0.008 N (Fisher, Ottawa, ON, Canada, >94%) diluted in water. The flow rate was set at 0.6 mL/min for 30 min. A volume of 5 µL of the sample was injected into the column.

### 4.9. Quantification of 2-Phenylethanol

The samples were thawed up at 4 °C for analysis. HPLC (Agilent Technologies 1230 infinity II, USA) coupled with a UV-visible forward optical scanning detector was used to determine the concentration of 2PE in culture broth samples. Pure 2PE (Sigma-Aldrich Burlington, MA, USA, >98%) was used as standard for the calibration curve. The HPLC was equipped with a ZORBAX Eclipse XDB-C18 (Agilent Technologies Inc., USA) column with a length of 250 mm, an internal diameter of 4.6 mm, and a film thickness of 5 µm. A mobile phase of water:methanol (50:50) was used at a flow rate of 0.5 mL/min for 30 min and the detection wavelength was 370 nm. The temperature of the column was set at 30 °C and the volume of injection was 10 µL.

### 4.10. Statistical Analysis

For the first step of this study, a one-way ANOVA (*p*-value < 0.05) with the Tukey test as a post-hoc test was performed in R core team 2020 software. The analysis was used to study the effect of the addition of hydrolyzed and nonhydrolyzed agri-food residues to the base whey medium on 2PE production. The independent variable was the type of culture media (WNC, WNS, WNBSY, WHC, WHS, and WHBSY) using WM as control, and the dependent variable was the concentration of 2PE at the end of the fermentation.

For the second step of the study, a one-way ANOVA (*p*-value < 0.05) with the Tukey test as a post-hoc test was performed in R core team 2020 software. The analysis was used to study the effect of WM and WBSY fermentation medium on 2PE production. The independent variable was the medium type, whereas the dependent variable was the concentration of 2PE at the end of the fermentation.

## 5. Conclusions

The treatment and valorization of agri-food residues was studied by means of fermentation using *Kluyveromyces marxianus* and *Debaryomyces hansenii* under co-culture mode. Both hydrolyzed and nonhydrolyzed agri-food residues were used as nitrogen sources for the growth of the yeasts. The high content of Cl^−^ and K^+^ present in the hydrolyzed residues inhibited the growth of *K. marxianus*. *D.hansenii* showed an adaptation to the osmotic stress caused by these ions but the production of 2PE was negligible. Among the agri-food residues tested, brewer’s spent yeast (BSY) was an excellent source of nitrogen, allowing for the highest 2PE yield for the fermentation assays in flasks. The controlled aeration in bioreactors and the use of BSY as a supplement to whey produced high consumption of L-Phe and productivity of 2PE. The co-fermentation of two agri-food residues (whey and BSY) produced better results than those reported in other studies on whey valorization into 2PE. In addition, the liquid effluent of fermentation presented COD content for agri-food residues under the Canadian regulatory limits of discharge. Preliminary economic analysis of a fermentation method using agri-food residues as raw materials for yeast fermentation in comparison to using yeast extract and peptone showed a significant saving of culture media cost of 82% when the nitrogen source was provided when using an agri-food residue instead of a traditional culture medium. This highlights the potential of agri-food co-fermentation using yeasts as a valorization process to produce high value-added biomolecules by co-fermentation with yeasts in co-culture mode and zero waste emissions for liquid fraction.

## Figures and Tables

**Table 1:** Chemical characterization of agri-food residues.

Parameters	Agri-Food Residues			
	Cheese Whey *	Crab Headshells	Residual Soy Cake	Brewer’s Spent Yeast
pH	4.88 ± 0.02	N.A.	N.A.	5.38 ± 0.01
Lactose (g/kg_residue_)	35.44 ± 0.00	N.A.	N.A.	N.A.
Total organic carbon (g/kg_residue_)	17.18 ± 0.25	N.A.	N.A.	63.79 ± 0.30
Total nitrogen Kjeldahl (g/kg_residue_)	0.93 ± 0.05	52.18 ± 0.01	9.30 ± 0.04	13.12 ± 1.41
Total solids (g/kg_residue_)	48.43 ± 0.07	592.30 ± 0.01	209.80 ± 0.22	220.25 ± 13.12
Ashes (g/kg_residue_)	3.62 ± 0.08	407.70 ± 0.01	6.20 ± 0.15	4.20 ± 0.10
Chemical oxygen demand (gO_2_/kg_residue_)	64.94 ± 2.93	430.46 ± 17.96	296.41 ± 14.41	320.50 ± 2.00
Total protein (g/kg_residue_)	5.71 ± 0.37	180.21 ± 6.61	85.60 ± 5.85	72.65 ± 0.99
L-phenylalanine (g/kg_residue_)	3.00 ± 0.01	10.00 ± 3.00	5.00 ± 0.10	11.01 ± 0.02
Valine (g/kg_residue_)	0.00 ± 0.00	0.00 ± 0.00	0.02 ± 0.00	2.00 ± 0.01
Methionine (g/kg_residue_)	12.00 ± 0.00	0.00 ± 0.00	0.00 ± 0.00	1.00 ± 0.01
Isoleucine (g/kg_residue_)	0.00 ± 0.00	2.00 ± 0.10	4.01 ± 0.01	0.00 ± 0.00
Leucine (g/kg_residue_)	5.02 ± 0.01	1.01 ± 0.02	1.02 ± 0.01	2.15 ± 0.00
Tryptophan (g/kg_residue_)	4.01 ± 0.20	5.05 ± 0.01	4.01 ± 0.09	9.10 ± 0.03
Calcium as Ca^2+^ (g/kg_residue_)	0.27 ± 0.01	129.00 ± 0.03	0.54 ± 0.01	0.32 ± 0.02
Potassium as K^+^ (g/kg_residue_)	0.11 ± 0.01	3.47 ± 0.01	1.77 ± 0.02	3.10 ± 0.01
Magnesium as Mg^2+^ (g/kg_residue_)	0.06 ± 0.00	8.02 ± 0.01	0.25 ± 0.01	0.58 ± 0.01
Sodium as Na^+^ (g/kg_residue_)	0.37 ± 0.00	9.06 ± 0.03	0.05 ± 0.00	0.02 ± 0.00

N.A. = not applicable. * The determined density of whey (1030 g/L_whey_) was used to calculate the content of each parameter.

**Table 2:** Overall performance of yeasts during the fermentation assays for whey media enriched with agri-food residues at flask scale.

Parameter	Fermentation Conditions						
	WM Control	WNC	WHC	WNS	WHS	WNBSY	WHBSY
Lactose consumption rate (g_lacrose_/L·h)_24 h_	0.84 ± 0.00	0.17 ± 0.00	0.12 ± 0.01	0.40 ± 0.00	0.11 ± 0.00	0.83 ± 0.01	0.12 ± 0.01
Ethanol productivity (g_ethanol_/L·h)_24 h_	0.24 ± 0.01	0.00 ± 0.00	0.00 ± 0.00	0.22 ± 0.01	0.00 ± 0.00	0.30 ± 0.01	0.00 ± 0.00
Ethanol yield production (g_ethanol_/g_lactose_)_24 h_	0.23 ± 0.02	0.00 ± 0.00	0.00 ± 0.00	0.39 ± 0.02	0.00 ± 0.00	0.25 ± 0.01	0.00 ± 0.00
L-phenylalanine concentration (g_L-Phe_/L)_0 h_	2.99 ± 0.06	0.46 ± 0.00	0.45 ± 0.01	0.43 ± 0.01	1.08 ± 0.00	0.54 ± 0.01	0.41 ± 0.00
L-phenylalanine concentration (g_L-Phe_/L)_48 h_	2.24 ± 0.09	0.41 ± 0.01	0.42 ± 0.01	0.02 ± 0.00	1.29 ± 0.00	0.42 ± 0.00	0.48 ± 0.04
L-phenylalanine consumption rate (mg_L-Phe_/L·h)_48 h_	15.75 ± 0.65	1.70 ± 0.10	5.65 ± 0.55	8.55 ± 0.10	0.40 ± 0.10	1.85 ± 0.15	2.80 ± 0.20
2-phenylethanol concentration (g_2PE_/L)_48 h_	0.67 ± 0.05	0.01 ± 0.00	3.50 × 10^−4^ ± 0.50 × 10^−4^	0.18 ± 0.00	2.00 × 10^−4^ ± 0.10 × 10^−4^	0.16 ± 0.00	0.00 ± 0.00
2-phenylethanol productivity (mg_2PE_/L·h)_48 h_	17.00 ± 1.00	0.65 ± 0.04	0.04 ± 0.00	7.60 ± 0.00	0.08 ± 0.00	3.35 ± 0.05	0.00 ± 0.00
2-phenylethanol yield production (g_2PE_/g_L-Phe_)_48 h_	0.89 ± 0.01	0.40 ± 0.01	0.34 ± 0.00	0.46 ± 0.01	0.00 ± 0.00	2.44 ± 0.10 *	0.00 ± 0.00

**WM**: whey medium fermentation used as control and comparative reference of the other media; **WNC:** whey medium supplemented with nonhydrolyzed crab; **WHC**: whey medium supplemented with hydrolyzed crab; **WNS:** whey medium supplemented with nonhydrolyzed residual soy cake; **WHS:** whey medium supplemented with hydrolyzed residual soy cake; **WNBSY:** whey medium supplemented with nonhydrolyzed brewer’s spent yeast; **WHBSY**: whey medium supplemented with hydrolyzed brewer’s spent yeast. For the calculation of each kinetics parameter, the considered fermentation time is indicated. * Apparent yield (see explanation in text).

**Table 3:** Overall performance of yeasts during the fermentation assays for WM and WBSY media in the 2 L bioreactors.

Parameter	Fermentation Conditions	
	WM	WBSY
Initial lactose concentration (g/L)	19.7 ± 0.3	19.6 ± 0.1
Initial L-phenylalanine concentration (g/L)	3.4 ± 0.2	3.2 ± 0.5
Initial brewer’s spent yeast mass (g/L)	0.0 ± 0.0	61.1 ± 0.0
Initial total organic carbon (g/L)	17.6 ± 0.5	12.6 ± 0.2
Initial total nitrogen (g/L)	3.6 ± 0.3	2.2 ± 0.1
Initial C/N ratio	4.9 ± 0.3	5.5 ± 0.1
Initial pH	6.5 ± 0.0	6.5 ± 0.0
Lactose consumption rate (g_lactose_/L·h) _24 h_	0.8 ± 0.0	0.8 ± 0.0
Ethanol productivity (g_ethanol_/L·h)_8 h_	0.1 ± 0.0	0.3 ± 0.0
Ethanol yield production (g_ethanol_/g_lactose_)_8 h_	0.3 ± 0.0	0.7 ± 0.0
L-phenylalanine concentration (g_L-Phe_/L)_31 h_	1.3 ± 0.0	0.0 ± 0.0
L-phenylalanine consumption rate (g_L-Phe_/L·h)_31 h_	0.06 ± 0.00	0.1 ± 0.0
2-phenylethanol concentration (g_2PE_/L)	0.7 ± 0.1_31 h_	1.8 ± 0.0_48 h_
2-phenylethanol productivity (g_2PE_/L·h)	0.03 ± 0.00_31 h_	0.04 ± 0.0_48 h_
2-phenylethanol yield production (g_2PE_/g_L-Phe_)	0.4 ± 0.0_31 h_	0.6 ± 0.0_48 h_

Subindices near the values of each kinetics parameter correspond to the time of fermentation at which they were calculated. **WM**: whey medium fermentation used as control and comparative reference of the other media. **WBSY:** whey medium enriched with brewer’s spent yeast. For the calculation of each kinetics parameter, the considered fermentation time is indicated.

## Supplementary Materials

The following supporting information can be downloaded at https://www.mdpi.com/article/10.3390/molecules28145536/s1; supporting information can be found in the following supplementary materials: Table S1: One-way ANOVA of the effect of agri-food residue fermentation using a co-culture of yeast in 2-phenylethanol production. The treatments comprise fermentation of nonhydrolyzed and hydrolyzed agri-food residues. Table S2: One-way ANOVA of the effect of agri-food residue co-fermentation in bioreactors using a co-culture of yeast in 2-phenylethanol production. The treatments are the fermentation of whey medium and whey enriched with brewer’s spent yeast as co-substrate. Table S3: Initial and final conditions during the fermentation assays for WM and WBSY media in the 2 L bioreactors. Also included are the considerations and calculations for the economic analysis and the corresponding Table S4: Economic comparison of yeast fermentation of 1000 L using pure reagents and agri-food residues for 2-phenylethanol production.
