# Supplementary material for: Co-Fermentation of Agri-Food Residues Using a Co-Culture of Yeasts as a New Bioprocess to Produce 2-Phenylethanol
Source: Molecules. 2023 Jul 20;28(14):5536. doi: 10.3390/molecules28145536 (PMC10385721; doi:10.3390/molecules28145536)
Supplement: Supplementary file 1 [file molecules-28-05536-s001.zip › molecules-2498708-supplementary.pdf]

Table S1. One-way ANOVA of the effect of agri-food residues fermentation using a co-culture of yeasts on 2-phenylethanol production. The treatments comprise fermentation of non-hydrolyzed and hydrolyzed agri-food residues.

Source of variation	Degree of freedom	$\sum Squares$	$Mean\ squares$	F - value	p – value
Type of medium	6	1.088	0.181	469	2.6×10^{-5}*
Residuals	14	0.005	0.000		

Treatments	Diff	Lwr	Upr	p – value adjusted
WHBSY-WM	-0.675	-0.730	-0.620	0.000*
WHC-WM	-0.675	-0.729	-0.620	0.000*
WHS-WM	-0.675	-0.729	-0.620	0.000*
WNBSY-WM	-0.514	-0.568	-0.459	0.000*
WNC-WM	-0.662	-0.717	-0.607	0.000*
WNS-WM	-0.493	-0.547	-0.438	0.000*
WHC-WHBSY	0.000	-0.054	0.055	1.000
WHS-WHBSY	0.000	-0.055	0.055	1.000
WNBSY-WHBSY	0.161	0.106	0.216	0.000*
WNC-WHBSY	0.013	-0.042	0.068	0.980
WNS-WHBSY	0.182	0.128	0.237	0.000*
WHS-WHC	0.000	-0.055	0.055	1.000
WNBSY-WHC	0.161	0.106	0.216	0.000*
WNC-WHC	0.013	-0.042	0.067	0.983
WNS-WHC	0.182	0.127	0.237	0.000*
WNB-WHS	0.161	0.106	0.216	0.000*
WNC-WHS	0.013	-0.042	0.068	0.982
WNS-WHS	0.182	0.127	0.237	0.000*
WNC-WNBSY	-0.148	-0.203	-0.094	0.000*
WNS-WNBSY	0.021	-0.034	0.076	0.836
WNS-WNC	0.169	0.115	0.224	0.000*

*Significant at 5% level ($p \leq 0.05$). **WM**: whey medium fermentation which is used as control and comparative reference of the other media; **WNBSY**: whey enriched with non-hydrolyzed brewer's spent yeast; **WNC:** whey enriched with non-hydrolyzed crab residues; **WNS**: whey enriched with non-hydrolyzed residual soy cake; **WHBSY**: whey enriched with hydrolyzed brewer's spent yeast; **WHC:** whey enriched with hydrolyzed crab residues; **WHS**: whey enriched with hydrolyzed residual soy cake; **Diff**: the difference between means of the two groups; **Lwr, Upr**: the lower and the upper endpoint of the confidence interval at 95% (default); and ***p*-value adjusted:** p-value after adjustment for the multiple comparisons.

Table S2. One-way ANOVA of the effect of agri-food residues co-fermentation in bioreactors using a co-culture of yeasts on 2-phenylethanol production. The treatments are the fermentation of whey medium and whey enriched with brewer's spent yeast as co-substrate.

Source of variation	Degree of freedom	$\sum Squares$	*Mean squares*	***F*** **- value**	***p* – value**
Type of medium	1	2.72	2.72	8.16	0.0083*
Residuals	26	8.65	0.33		
**treatments**	**Diff**	**Lwr**	**Upr**	***p*-value adjusted**	
WM-WBSY	-0.6229	-1.071052	-0.1747482	0.0083031*	

*Significant at 5% level ($p \leq 0.05$). **WM**: whey medium fermentation which is used as control and comparative reference of the other media; **WBSY**: whey enriched with brewer's spent yeast and L-Phenylalanine. **Diff**: difference between means of the two groups; **Lwr, Upr**: the lower and the upper-end point of the confidence interval at 95% (default); and ***p*-value adjusted:** p-value after adjustment for the multiple comparisons.

Table S3. Initial and final conditions during the fermentation assays for WM and WBSY media in the 2 L bioreactors.

Parameter	Fermentation			
	Initial conditions		Final conditions	
	WM	WBSY	WM	WBSY
Total chemical oxygen demand (gO_2/L)	39.3 ± 1.4	36.6 ± 1.5	20.8 ± 0.8	6.9 ± 0.2
Soluble chemical oxygen demand (gO_2/L)	26.0 ± 0.5	25.1 ± 0.2	0.0 ± 0.0	0.0 ± 0.0
Cl^- (g/L)	3.4 ± 0.1	0.3 ± 0.0	6.6 ± 0.0	2.4 ± 0.0
K^+ (g/L)	1.3 ± 0.0	1.1 ± 0.0	2.6 ± 0.0	2.8 ± 0.0
Na^+ (g/L)	2.5 ± 0.1	0.2 ± 0.0	3.3 ± 0.0	0.1 ± 0.0
Mg^{2+} (mg/L)	30.0 ± 1.5	75.0 ± 3.7	32 ± 0.3	74.0 ± 0.7

*Under limits of detection

An **economic comparison** of two culture media for whey fermentation using yeasts under co-culture mode was done considering a basis of 1000 L as follows:

1. A culture medium which was composed of whey diluted with water in a proportion of 1:0.8 to have the initial concentration of lactose of 20 g/L, yeast extract 10 g/L and peptone 20 g/L (equivalent to 3.7 g of N/L), and L-Phenylalanine (L-Phe) 3 g/L.
2. A culture medium which was composed of whey diluted with water in a ratio of 1:0.8 to have the initial concentration of 20 g/L of lactose, brewer's spent yeast (BSY) 61.11 g/L (equivalent to 3.7 g of N/L), and L-Phe 3 g/L.

Then the difference of both culture media was basically the nitrogen source. The whey and the BSY were residues issued by local enterprises in Quebec province, a cheese producer and a microbrewery, respectively. Currently, both enterprises do not valorize these residues, they sent them to waste treatment units. For the economic comparison were considered only the raw materials, commercially available in Canada and residues which have the potential to be valorized. For this analysis, the fermentation factory was supposed to be close to the cheese factory, this was manifested by a third enterprise which is interested to valorize the whey. This localization was chosen because of the proximity to the main raw material needed for fermentation, this means that the transport of whey is minimum, maybe only by pumping.

The supplementary material ST4 shows the type and mass of raw materials used for fermentation in a culture media basis of 1 000 L. The ST4 also shows the sources of raw materials. In the case of raw materials that must be purchased, wholesale distributors and prices were considered. According to the composition of each culture media, the cost of culture medium using yeast extract and peptone as N source is 658 US$, whereas for the BSY as nitrogen source it is 118 US$. This corresponds to save 82.04 % when the agri-food residue is used.

In order to complete the economic comparison of culture media, the price of the main product in wholesale format and nature certification was considered. Currently, 1 kg of 2PE produced by natural methods, such as fermentation or extraction from plants, has a market price of 640 US$. Regarding the mass of 2PE that could be produced in 1000 L of culture medium, according to concentrations observed in the present study, using pure reagents it is possible to recover 0.7 kg of 2PE, equivalent to an income of 448 US$, which is lower than the cost of buying the raw materials. Whereas, with the agri-food residue, the mass of 2PE that can be recovered is 1.8 kg for an income of 1 152 US$. This is much higher than the cost of raw materials of 118 US$. This difference shows a real potential of using the agri-food residue as nitrogen source. The profit of 1 033 US$ can cover the fees of transport of L-Phe and the agri-food residues, specially the BSY which is needed in lower quantity than the whey, as well as the operation costs. The increase of scale would increase the profits, showing that the valorisation of residues has great potential in terms of developing sustainable development with economic suitability in rural regions.

Table S4. Economic comparison of whey fermentation of 1000 L using pure reagents and agri-food residues for 2-phenylethanol production.

Fermentation conditions				Wholesale information				Price of raw materials for fermentation of 1000 L	
Parameter	Unity	Value laboratory scale	Value pilot scale	Price (US$/ kg)	Enterprise	Web-Site	Consulting date	Considering the use of pure reagents	Considering the use of agri-food residues
Working volume	L	1.8	1000	NA	NA	NA	NA	NA	NA
Yeast extract	kg	0.02	10.00	8.00	Selina Wamucii	A	07-12-2023	$10\ kg \cdot 8 \frac{\$}{kg} = 80\ \$$	0
L-Phe	kg	0.01	3.00	39.41	BoxNutra	B	07-13-2023	$3\ kg \cdot 39.41 \frac{\$}{kg} = 118.25\ \$$	$3\ kg \cdot 39.41 \frac{\$}{kg} = 118.25\ \$$
Peptone	kg	0.04	20.00	23	Vita Plus	C	07-12-2023	$20\ kg \cdot 23 \frac{\$}{kg} = 460\ \$$	0
Cheese Whey	kg	1.03	286.11	0	Local cheese factory	NA	07-12-2023	0	0
BSY	kg	0.11	61.11	0	Local microbrewer	NA	07-12-2023	0	0
2PE produced	kg	0.7	1.8	640	Vigon	D	07-12-2023	NA	NA
**Overall price of 1000 L fermentation**								**658.25 US$**	**118.25 US$**

NA: not applicable; A: https://www.selinawamucii.com/insights/prices/canada/yeast/#market-insights; B: https://boxnutra.com/products/l-phenylalanine?variant=43572381745376; C: https://vita-plus.ca/products/vrac-caseine; D: https://www.vigon.com/product/phenyl-ethyl-alcohol-natural/.
